# Self-care of long-term conditions: patients’ perspectives and their (limited) use of community pharmacies

**DOI:** 10.1007/s11096-016-0418-y

**Published:** 2017-01-24

**Authors:** Oladapo J. Ogunbayo, Ellen I. Schafheutle, Christopher Cutts, Peter R. Noyce

**Affiliations:** 1 Centre for Pharmacy Workforce Studies (CPWS), Division of Pharmacy and Optometry, School of Health Sciences, Faculty of Biology, Medicine and Health, The University of Manchester, Stopford building, Oxford Road, Manchester, M13 9PT UK; 2 Centre for Pharmacy Postgraduate Education (CPPE), Division of Pharmacy and Optometry, School of Health Sciences, Faculty of Biology, Medicine and Health, The University of Manchester, Stopford building, Oxford Road, Manchester, M13 9PT UK

**Keywords:** Community pharmacy, Healthcare professionals, Long-term conditions, Patient perspective, Qualitative interviews, Self-care, United Kingdom

## Abstract

*Background* Self-care support is an ‘inseparable’ component of quality healthcare for long-term conditions (LTCs). Evidence of how patients view and use community pharmacy (CP) to engage in self-care of LTCs is limited. *Objective* To explore patients’ perspectives of engaging in self-care and use of CP for self-care support. *Setting* England and Scotland. *Method* Qualitative design employing semi-structured interviews. LTCs patients were recruited via general practitioners (GPs) and CPs. Interviews were conducted between May 2013 and June 2014; they were audio-recorded, transcribed verbatim and analysed thematically. *Results* Twenty-four participants were interviewed. Three main themes emerged: engaging in self-care, resources for self-care support and (limited) use of community pharmacy. Participants’ LTC ‘lived experience’ showed that self-care was integral to daily living from being diagnosed to long-term maintenance of health/wellbeing; self-care engagement was very personal and diverse and was based on beliefs and experiences. Healthcare professionals were viewed as providing information which was considered passive and insufficient in helping behavioural change. Non-healthcare sources (family, carers, friends, internet) were important in filling active support gaps, particularly lifestyle management. Participants’ use of, and identified need for, community pharmacy as a resource for self-care support of LTCs was limited and primarily focussed on medicines supply. There was low awareness and visibility of CPs’ potential roles and capability. *Conclusion* CP needs to reflect on patients’ low awareness of its expertise and services to contribute to self-care support of LTCs. Rethinking how interventions are designed and ‘marketed’; incorporation of patients’ perspectives and collaboration with others, particularly GPs, could prove beneficial.

## Impact of findings on practice

- Patients’ low awareness and limited use of community pharmacy as a self-care resource may be indicative of community pharmacists’ professional identity as ‘dispensers’ of medicines.
- The ‘patient voice’, often ‘unheard’ in community pharmacy research, needs to be recognised and incorporated into new and existing community pharmacy research, interventions and service design.
- Community pharmacy needs to demonstrate evidence of its value in the management and self-care support of LTCs, alongside effectively ‘marketing’ this value to patients.

## Introduction

Long-term conditions (LTCs) like diabetes, cardiovascular diseases (CVD), chronic respiratory diseases and cancers are now recognised as the greatest challenge facing twenty-first century healthcare [1]. This recognition has led many governments, policymakers and researchers globally to develop strategies to efficiently manage limited healthcare resources to meet future demands of people with LTCs. In the United Kingdom (UK), healthcare professionals working in the National Health Service (NHS) have been encouraged to develop evidence of effective models of care that improve the effectiveness and cost-effectiveness of healthcare for people with LTCs [2, 3]. Self-care support (also self-management support) is one model of care that has been recognised as a potential paradigm shift in how healthcare is provided to people with LTCs as it aims to empower and support patients to take control and enable them to self-care for their own health and well-being [4]. The UK government’s NHS Five-Year Forward View [5] published in 2014 makes a strong case for patients, particularly those with LTCs, to be given more control of their own care. There is ample evidence that self-care support works [6] and because of these well-documented benefits in improving patient outcomes and being resource-efficient [7–10], health policy and research now place great emphasis on healthcare adopting the principles of self-care support in their routine practice.

Self-care support in primary care has been described as an *inseparable* component of high quality healthcare for LTCs [11]. It is an essential part of the chronic care model (CCM) [12] which emphasises active engagement and empowerment of patients before, during and after consultations with healthcare professionals [13–15]. Self-care support is now regarded as a distinct model of care for LTCs but there is some ambiguity in the main components of a clinically effective programme for self-care support [11]. What is however clear is that self-care support of LTCs in primary care is multifaceted, with research evidence suggesting that multilevel interventions such as those that target healthcare professionals, patients and organisational structures at the same time are more effective than those that target simple or single components [16]. Self-care support requires a fundamental shift in the healthcare professional-patient consultation with patient-centred interactions that involve shared decision making, personalised care planning, goalsetting, and proactive follow-up [17].

Self-care is an activity of daily living for people, whether healthy or with a LTC, and ranges from simple activities to promote health such as exercising and eating healthily, to more complex actions to restore health such as receiving medical treatment and rehabilitation activities [16]. Patients with LTCs engage in self-care activities to achieve normality in their everyday lives, maintain social relationships and participate in meaningful activities in the community [18–20]. Examples of these activities include lifestyle modification such as stopping smoking, taking medications, self/symptom-monitoring and seeking more information and support about living with LTCs [4, 16]. Factors such as personal and lifestyle characteristics, health status, resources, environmental factors and the healthcare system affects how people engage in self-care [21]. Patients will often seek support for self-care from both healthcare and non-healthcare sources based on their instrumental, psychosocial and relational needs [22]. Studies have shown that non-healthcare sources (family, friends, peers) play a more significant role in supporting many aspects of self-care including emotional and lifestyle support, although patients still rely on healthcare professionals for support with the medical aspect of their LTCs [18, 20, 23]. Among healthcare professionals, community pharmacy teams are least considered for ongoing support although the reasons for this is not clearly evident [23].

Community pharmacy is often described as the healthcare profession that is most accessible to patients with LTCs when collecting prescribed medicines [24–27]. Community pharmacy is also available to offer advice to patients who need help and to signpost them to other support services. Internationally, community pharmacy’s contribution to the ongoing care of LTCs is largely restricted by a focus on dispensing-related reimbursement mechanisms which stifles the recognition and incentivisation of other support activities that community pharmacists could provide [28]. In 2005, community pharmacy in the UK moved to more innovative remuneration models that reimburse community pharmacists for providing a whole range of services [29]. For patients with LTCs, these include public health services such as healthy living advice and support for lifestyle changes, and medicines-related services which aim to help improve patients’ medicines knowledge, understanding and adherence [29]. Medicines-related services, the English Medicines Use Review (MUR) and New Medicine Service (NMS) and the Scottish Chronic Medication Service (CMS), have, however, been criticised as focussed on quantity rather than quality [30, 31]. A study exploring community pharmacy’s views on, and contributions to, holistic self-care support of LTCs suggests a medicines-focussed approach, rather than a patient-centred one [32]. Currently, the evidence of the impact of community pharmacy’s roles in improving patient outcomes and reducing healthcare utilisation remain inconclusive and ineffective in influencing governments [33].

The majority of the research literature on the management of LTCs in community pharmacy describes interventions that target specific patient outcomes such as medicines adherence [34], improvements in quality of life [35] and uptake/utilisation of services [36, 37]. The views and perspectives of patients with LTCs are usually incorporated in research that focus on interventions such as specific services [30, 38], LTCs-specific intervention [39, 40], medicines management services [41, 42] adherence-improving interventions [43], and lifestyle and public health interventions [44, 45]. However, these focus on patients’ preferences, experiences and satisfaction with these services and interventions. While many of these studies generally report favourable patient perspectives, experience and satisfaction, there are few qualitative studies that have examined a holistic perspective of how patients with LTCs view and utilise community pharmacy for their LTCs in their everyday lives. Indeed, qualitative research that has explored patients’ perspectives of the holistic care that they receive from healthcare professionals in general is also limited [46–49]. The few studies that exist are usually driven by agendas and priorities set by healthcare professionals, which may lead to a mismatch between the research undertaken and the actual needs of patients [50, 51]. Patients’ needs are paramount in any research that recommends changes to services or interventions, and incorporating the views and perspectives of patients has been described as morally desirable as well as having the potential to improve the intervention [49, 52].

## Aim

The overall aim of this study was to explore patients’ perspectives of the current and potential contributions of community pharmacy to self-care support of LTCs. The objectives were:

- To explore and describe patients’ perspectives of how they engage in self-care of LTCs and the resources they access for self-care support.
- To understand how patients view and use community pharmacy in the ongoing management of their LTCs for self-care support.

## Ethics approval

The study received NHS Research Ethics Committee and local NHS Research and Development approvals in England and Scotland.

## Method

This study employed a qualitative research design, underpinned by descriptive phenomenology [53] which focusses on understanding the ‘lived experience’ of people from a first person point of view through the interaction of the researcher and the participants [54]. Participants were patients living with LTCs in England and Scotland, and included people that had at least one of diabetes mellitus (type 1 and 2), chronic respiratory diseases (asthma; COPD) and cardiovascular diseases (hypertension; hypercholesterolemia; heart conditions). Participants were sampled conveniently and purposively to allow for maximal variation [55] in the type of LTCs and demographic characteristics (age, gender, ethnicity, deprivation, education).

Participants were recruited between May 2013 and June 2014. The initial recruitment strategy was to identify patients from their general practitioner (GP) practices. However, poor participation of GP practices led to a change of strategy to include recruiting patients by pharmacists. In England, one GP practice and ten community pharmacists supported patient recruitment; in Scotland one GP practice, two practice pharmacists and one community pharmacist helped with patient recruitment. The researcher attended GP diabetic and asthma clinics, where patients were approached and provided with the recruitment pack and verbal explanation of what the study entailed. The pharmacists identified and approached eligible patients and provided them with the same written and verbal study information. Interested patients provided their contact details and consent to the researcher directly (in the GP practices) or to the pharmacists, who passed these onto the researcher. Interested patients were contacted by telephone and/or email and the interview date and venue were arranged.

This study used semi-structured one-on-one interviewing of participants as its primary method of data collection. Participants also completed a pre-interview questionnaire collecting demographic data. The interview topic guide was developed from the literature and evolved iteratively as interviewing progressed. The topic guide focussed on extending current knowledge on patients’ self-care behaviours and activities and factors affecting their use of healthcare and non-healthcare sources for support. While previous studies have explored these topic areas in detail, this study focussed on building on these studies and gaining deeper insight into patients’ self-care behaviours in relation to their use of community pharmacy for self-care support. Hand written notes were taken during and after each interview to record any important observations, additional statements and the researcher’s reflections. Interviews were conducted face-to-face with participants, at their homes and in two cases at an alternative location (coffee shops). Interviews lasted between 15 and 40 min (average of 33 min). Following written/signed consent, all interviews were audio-recorded and transcribed verbatim.

Data analysis was undertaken thematically [56] underpinned by the philosophical stance of descriptive phenomenology [54], which is characterised by a reflection of an experience by the researcher based on the descriptions provided by participants. The researcher gathered concrete descriptions of specific experience from participants, adopted the attitude of ‘phenomenological reduction’ to understand the experience and sought to capture the ‘essential structure’ of the experience within the context of the participants [53]. The process of phenomenological reduction and capturing of the essential structure of entire and individual interviews undertaken in this study followed the steps described by Todres [53].

## Results

### Participants

Twenty-four patients were interviewed, 15 in England and nine in Scotland. Fifteen were female and participants’ mean age was 62 years (SD = 20.1, Range = 24–92 years). Participants’ ethnicity was predominantly White (n = 19), although Black (n = 3), Asian (n = 1) and other ethnic groups (Mixed race, n = 1) were also represented. Thirteen participants were educated up to high school level, the remaining 11 had higher education qualification. Seventeen participants were retired, two were unemployed, and the rest were in full-time or part-time work (n = 5). Most participants (n = 20) had multiple LTCs that included asthma/COPD (n = 11), cardiovascular diseases (n = 7) and diabetes (n = 6). The age at which participants had been diagnosed with their LTC(s) ranged from birth to 40 years, and they took an average of five regular medicines (range 1–24).

### Main findings

#### Engaging in self-care

Interviews explored participants’ ‘lived experience’ where it was found that self-care was ingrained in all stages of the LTC trajectory; from diagnosis, through the acute management of physical and emotional aspects, to long-term maintenance of health and wellbeing. Table 1 summarises some of the key themes on how participants engaged in self-care, following this trajectory and illustrated with exemplar quotes. Participants described self-care as a complex range of behaviours and activities which were diverse and shaped by their individual illness experiences, beliefs and personal circumstances. For example, one participant described how he adjusted his drinking lifestyle based on information he had previously received and his experience of self-monitoring his condition. While participants’ sources of support to engage in self-care varied and were driven by variable factors, it was generally agreed that there were unmet self-care support needs along their LTC trajectory, particularly in relation to managing emotions and lifestyle behaviours (see Table 1).

**Table 1:** Engaging in self-care—broad and specific themes and illustrative quotes

Broad themes—aspects of self-care along LTC trajectory	Specific themes: engaging in a self-care activity	Exemplar quotes
Being diagnosed with a LTC	Seeking information after being diagnosed	I was in hospital for an asthma attack, a really bad asthma attack and it took ages to get over it and the doctors there said I had COPD but I didn’t know what it was then so I had a look myself what it was. (EP12 – 70 year old female with COPD and asthma)
	Finding meaning and adjusting lifestyle to LTC	Yeah, when I was first diagnosed I pretty much stopped drinking completely because I was kind of misinformed really but now I started drinking a lot more, and I just monitor it. (EP4 – 26 year old male with diabetes)
Managing physical health aspect of LTC	Taking/adhering to prescribed medications as a habit/ritual	Taking your medications, well, that just comes automatic now. Once you’ve been taking it for years its habit now, its habit and you know exactly what you’re doing. (SP4 – 77 year old male with asthma, CVD and arthritis)
	Changing/modifying lifestyle to cope with physical demands	Well, I mean in the past I used to be very active and played tennis and hockey, but I haven’t done that for a long time. My husband and I fished, that was our hobby, but nowadays my main exercise is the garden. (EP5 – 85 year old female with asthma, CVD and diabetes)
	Symptom monitoring and management	I was in the supermarket and I was feeling horrible [hypoglycaemic]….I bought one of those little orange juices. I wouldn’t normally because it does shake your blood sugar up, but I drank some of that and the pure juice, of course it shoots your blood sugar up. (SP2 – 65 year old female with diabetes and CVD)
Managing emotional health aspect of LTC	Staying positive and hopeful	I mean we all worry as we get older you know, we are getting nearer to the end you know [laughs]….But its, um, you know, when you see people that are sick, it’s upsetting, you know, um, when you are hoping that those kind of things don’t happen to you, but you know, nobody knows. (EP1 – 70 year old male with CVD and gout)
	Seeking psychological counselling and support	Yes, when I was going through university, I did actually see a counsellor because one or two things became too heavy to deal with I did see a counsellor about the emotional side, but I must have only had about four sessions before I realised I can deal with this on my own… (EP6 – 26 year old male with a heart defect)
	Managing stress	Well, sometimes it [blood sugar] goes high, like, stress makes it just go up a lot, but, because I was very paranoid of going to sleep with low blood sugar. So, yeah, I’d say that my good control came probably, like, about four years ago when I started to realise how important your health actually is and if you just pay a little bit of attention to it, then it can be fine. (EP2 – 26 year old female with diabetes)
Long-term maintenance of health and wellbeing	Making healthy lifestyle choices	I take the tablets and I try and take as much exercise as possible. I could do with losing more weight. I’ve stopped smoking a long, long time ago. Um, and am trying on having a reasonable diet, ye. (EP8 – 78 year old male with asthma and CVD)
	Self-monitoring	Well, I’ve a fair idea, you know your own body better than anybody else and I have a fair idea of when I feel out of sorts and something isn’t right and if there’s something bothering me I won’t wait too long before I go to the doctor if it’s worrying me. (SP9 – 73 year old female with CVD and COPD)
	Healthcare utilisation	Yes. Recently, maybe two or three months ago, I was having hypos which meant I was in the supermarket and I was holding on to the shelf, because I thought I was going to [faint]. So I made an appointment to see the nurse and she said, you definitely have to see the doctor. As far as she was concerned everything was fine apart from that. So the doctor changed my tablets. (SP2 – 65 year old female with diabetes and CVD)

#### Resources for self-care support

While describing their lived experience, participants were probed about the support and resources they accessed to engage in self-care. Participants identified healthcare and non-healthcare resources with most indicating they combined multiple sources. In the context of gaining the knowledge and understanding about their particular LTCs, most participants suggested they got more information from non-healthcare resources such as family/friends, reading books and websites, because some healthcare professionals provided insufficient information.

I think I pieced together bits of information; I probably did look online a little bit and then GPs and nurses and just got combined information…the education I got wasn’t that fantastic, to be honest, …I mean I think the doctor at the hospital should have given me more information, I was just sort of given a bag of insulin pads and just told to go away… So I sort of had to just learn it on my own what worked and what didn’t work really….But also actually a lot of stuff that I found out was from another diabetic person, like, one of my friends that I met, like, he was very helpful and very informative

EP4 (26 year old male with diabetes)

Most participants indicated that their prescribing healthcare professional (mainly GPs) provided them with medicines information and advice, although many also indicated that they read the patient information leaflets that came with the medicines. Despite obtaining their prescribed medicines from community pharmacy, they did not mention community pharmacy as a source of information on medicines use, except occasionally when they had been prescribed a new medicine. While participants affirmed that they were aware they could obtain medicines-related information from the pharmacy, they felt they did not have the need to do so since they already obtained this from GPs. Some participants admitted that they were unsure whether community pharmacy could deal with problems relating to the use of prescription medicines.

I: And when you had the issue why did you go back to the doctor, did you think about going to the chemist rather than going to the doctor’s?

R: No, I didn’t actually, no, because I didn’t think a chemist…well, I just didn’t think, that maybe a chemist couldn’t mess with my prescription, I would have to go to the doctor’s, wouldn’t I?

EP12 (70 year old female with COPD, diabetes and CVD)

With regards to making lifestyle changes, many participants indicated that they had been offered information and advice from healthcare professionals, primarily doctors and nurses in their GP practices/hospitals, about making lifestyle changes. However, most patients acknowledged that healthcare professionals provided lifestyle information and advice mostly passively rather than being more proactive in supporting behaviour change or referring them to other services which offered more proactive support. Again, most patients resorted to non-healthcare professional sources to help them make the lifestyle changes for their LTCs.

I wanted to lose weight anyway and that’s what I was doing before the asthma flared up last year [laugh] and then all of a sudden I couldn’t do anything. …The doctors were like, well, you know, swimming and stuff, do something gentle, but you don’t lose weight doing gentle exercise. I’ve got a personal trainer at the gym to help me.

EP7 (24 year old female with asthma)

#### Community pharmacy as a resource for self-care support

The majority of participants talked about the primary purpose of community pharmacy to them being the supply of medicines; mainly prescribed medicines for their LTCs but also over-the-counter (OTC) medicines for minor ailments. All participants were taking regular medicines for their LTCs and most indicated that they had an established supply system set-up with the community pharmacy. After participants discussed their use of community pharmacy for collecting their prescribed medicines, they were probed further to discuss any other reasons when they made use of, or interacted with, their community pharmacy for managing their LTCs. Most participants struggled to come up with anything and none of them mentioned any of the main LTCs-specific services such as the MURs, the NMS and the CMS or lifestyle interventions such as the smoking cessation and healthy lifestyle services. Some participants however indicated that they were aware that community pharmacy offered a range of services but indicated that they did not feel the need to use these.

I know the pharmacy offers a lot of services, in terms of, like, free checks for this, checks for that and checks for this, I’ve never felt the need to go in and see them, like, one of them is a free check for your diabetes risk, which I feel is a bit unnecessary and, yeah, I know they do offer advice on prescriptions, but I’ve never felt the urge to take advantage. So I know that they do offer a lot, but, for me, my first port of call would be my doctor. EP13 (45 year old female with diabetes and CVD)

Participants were probed further on their awareness and use of any services or interventions in the community pharmacy for self-care support of LTCs. While most participants indicated awareness of community pharmacy as a potential self-care resource, some routinely returned to their GP, and others simply did not consider the community pharmacist as an option. When probed about this, some participants indicated that it was because their GPs was their first point of call, while some others suggested that community pharmacy was not readily visible to them.

I don’t know, it’s just my perception, I’ve always just gone to the doctor’s for advice and never the pharmacist, and it’s just the way that it’s always been for me; I’ve never really thought to question it with them; I don’t really know why, to be honest.

SP1 (62 year old female with diabetes and CVD)

This topic was explored further with participants asked to discuss any situations when they had chosen to visit the pharmacy for anything relating to their LTCs and the use of their medicines, instead of going to their GPs. Most participants struggled to think of a situation like that and went on to indicate that they always sought the help of their GP if they had any concerns about their medicines or their LTCs. They suggested that they would not readily consider seeking help from community pharmacy unless they had run out of all other options. When asked for the reason for this, most participants could not come up with a clear reason but indicated that it could be because they viewed community pharmacy as a medicines supplier.

I: And then with the diabetes what sort of situation would want you to go to the pharmacy and ask to see the pharmacist?

R: I suppose I would go there if, say, there was a big waiting list at my doctor’s, I couldn’t easily get to my diabetic doctor; they [community pharmacy] would probably be like my third or fourth choice of someone to ask….. Yeah. I honestly, I don’t know why, but I never really thought of using them, I’d just seen them as the people who give me my medication.

EP4 (26 year old male with diabetes)

Some participants further admitted that, while they were aware that community pharmacists were suitably qualified to help them, they felt community pharmacists did not appear visible enough for them to be approached for help. They again confirmed their perspective of community pharmacy being that of a medicines supplier, which dissuaded them from viewing and utilising community pharmacy for other purposes.

I know they are qualified; it’s just that it’s not your immediate thought …But for some reason, I don’t know, the pharmacist um, you just forget that the pharmacist is actually there [laughs]. You know, because you go into the pharmacy and there are lots and lots of, mainly women, um, who are making up medications and so on, according to prescriptions, and you tend to forget that the pharmacy and not a dispenser, you know, yea.

EP3 (57 year old female with diabetes, hypocholesteraemia and psoriasis)

## Discussion

While previous research has explored patients’ lived experiences of their LTCs, [20, 57] their perspectives of self-care [58] and support needs [22], this study extends this knowledge in the pharmacy literature. Earlier work by the authors that explored community pharmacy’s contributions to self-care support of LTCs found that pharmacists conceptualised and operationalised self-care support from a medicines-focussed and opportunistic perspective, although there was the recognition of the potential to contribute to a more holistic approach [32, 59]. This study complements this earlier work and provides a more complete understanding by drawing on patients’ perspectives of community pharmacy’s contributions to self-care support of LTCs. The key findings from all studies highlight the gaps of low public/patient awareness, and use of, community pharmacy as a resource for self-care support of LTCs, as well as a need for a more comprehensive self-care support and LTCs strategy in community pharmacy if its potential are to be realised.

There are some limitations in this study which should be acknowledged. None of the participants had recently used a community pharmacy LTC-specific service (MUR, NMS or CMS), possibly resulting in an unbalanced perspective as people who use these services may have a different view of community pharmacy’s self-care support roles. Furthermore, participants’ interest in taking part in this study and their high levels of education may mean that they were already knowledgeable and more involved in their healthcare, and this may have skewed their perceptions of community pharmacy’s potential role in self-care support. The short duration of the interviews may also be considered a potential limitation. However, this study did not aim to duplicate previous research that has explored patients’ perspectives of illness and self-care, but focussed on views and use of community pharmacy as a self-care support resource.

Patients with LTCs encounter and interact with a wide range of healthcare and non-healthcare networks that shape how they engage in self-care [60]. Patients in this study suggested that while healthcare professionals provided self-care information and advice relating to their LTCs, use of prescribed medicines and lifestyle management, these were often provided didactically and passively. This meant that patients had to rely on and combine other resources for active support to engage in self-care, mainly non-healthcare resources that included personal communities, non-health professionals and voluntary and community groups [61]. Among healthcare professionals, community pharmacists did not feature as a potential resource for self-care, even with regards to the use of prescribed medicines that were dispensed by community pharmacies. Participants suggested that the supply of prescribed medicines was the primary reason for interacting with community pharmacy and despite the LTC support services and interventions available in community pharmacy they were either not aware or did not readily take advantage of these.

Patients in this study looked to their GPs to take the lead in the care of their LTCs and viewed them as the first point of call for support with their medicines and general management of their health. There is however increasing recognition that community pharmacists are well-suited to lead the care of patients with stable, uncomplicated LTCs (those on stable medications) [62, 63]. This case has been made stronger with government recognition that the current structure and organisation of the primary care workforce and pressures on general practices may not be able to cope with current and future healthcare demands [5]. Collaborative partnership between the multidisciplinary teams and patients is a necessity to improve the effectiveness and cost-effectiveness of healthcare [5]. Community pharmacy in the UK has, in recent times, consistently made a case for an extended clinical role in the ongoing management of LTCs [62–64]. Better integration between GPs and pharmacists is thus likely to be fundamental to the success of community pharmacy’s contribution to self-care support. Indeed, self-care support of LTCs interventions that have been shown to be effective commonly involve GP-community pharmacy collaboration [64, 65].

This integration however, has not yet been successfully achieved, with the main barriers identified as professional isolation and ‘shopkeeper’ image of community pharmacy, lack of information sharing and limited cooperation and support between community pharmacy and GPs [66–70]. There is a plethora of studies that have examined and recommended major reforms to the working relationships and interactions between community pharmacy and GPs [71–74]. Some of these recommendations include inter-professional education at both undergraduate and practitioner levels [75, 76]; regular communication to improve the flow of information; [74] incentivising to work collaboratively through joint contracts; [70] and establishing and expanding the roles and contributions of GP practice pharmacists [77]. Recently, pivotal steps have been taken towards integration of medical and pharmaceutical service delivery in primary care [78], and there is a proposal to further improve GP-community pharmacy integration by recommending the development and incentivisation of joint contractual frameworks [79].

Finally, the ‘patient voice’, the perspectives of patients, is an area that needs to be given further consideration if community pharmacy is to improve its contributions to self-care support of LTCs. NHS England recognises this as a key policy area and stated that it “will ensure that public, patient and carer voices are at the centre of our healthcare services, from planning to delivery” [80]. This will require a better understanding of the factors and determinants of patient self-care behaviours and the resources they access, and recognising and incorporating patients’ perspectives of how they want to be supported to enhance their abilities to engage in self-care [9]. While patients in this study suggested that community pharmacy currently plays a limited role in self-care support of LTCs, this finding may be more closely linked to patients’ low awareness and lack of recognition of community pharmacists’ potential as a clinical healthcare professionals that could manage and support LTCs [23]. Furthermore, studies have shown that community pharmacists view themselves primarily as “dispensers of medicines” as their main professional role [81], which may reflect the lens that patients also use to view them. Indeed, the perspectives of patients in this study was that the primary role of community pharmacy was that of medicine supplier. Hence community pharmacy could do more to promote its professional image and what it could offer.

## Conclusion

Community pharmacy remains an untapped resource for self-care support of LTCs. This can be attributed to low awareness and uptake of community pharmacy as a resource by LTC patients. Community pharmacy may need to demonstrate evidence of its value in the management and self-care support of LTCs, alongside effectively ‘marketing’ this value to patients, healthcare professionals and the public.
